# Nafamostat Mesilate Enhances the Radiosensitivity and Reduces the Radiation-Induced Invasive Ability of Colorectal Cancer Cells

**DOI:** 10.3390/cancers10100386

**Published:** 2018-10-17

**Authors:** Hiroshi Sugano, Yoshihiro Shirai, Takashi Horiuchi, Nobuhiro Saito, Yohta Shimada, Ken Eto, Tadashi Uwagawa, Toya Ohashi, Katsuhiko Yanaga

**Affiliations:** 1 Department of Surgery, The Jikei University School of Medicine, 3-25-8, Nishi-Shinbashi, Minato-ku, Tokyo 105-8461, Japan; shirai@jikei.ac.jp (Y.S.); horiuchi@jikei.ac.jp (T.H.); h24dr-saito@jikei.ac.jp (N.S.); etoken@jikei.ac.jp (K.E.); uwatadashi@msn.com (T.U.); kyanaga@jikei.ac.jp (K.Y.); 2 Division of Gene Therapy, Research Center for Medical Science, The Jikei University School of Medicine, 3-25-8, Nishi-Shinbashi, Minato-ku, Tokyo 105-8461, Japan; shimada_y@jikei.ac.jp (Y.S.); tohashi@jikei.ac.jp (T.O.); 3 Division of Medical Oncology and Hematology, Department of Internal Medicine, The Jikei University School of Medicine, 3-25-8, Nishi-Shinbashi, Minato-ku, Tokyo 105-8461, Japan

**Keywords:** nafamostat mesilate, colorectal cancer, ionizing radiation, NF-κB, matrix metalloproteinases

## Abstract

Neoadjuvant chemoradiotherapy followed by radical surgery is the standard treatment for patients with locally advanced low rectal cancer. However, several studies have reported that ionizing radiation (IR) activates nuclear factor kappa B (NF-κB) that causes radioresistance and induces matrix metalloproteinase (MMP)-2/-9, which promote tumor migration and invasion. Nafamostat mesilate (FUT175), a synthetic serine protease inhibitor, enhances the chemosensitivity to cytotoxic agents in digestive system cancer cells by inhibiting NF-κB activation. Therefore, we evaluated the combined effect of IR and FUT175 on cell proliferation, migration and invasion of colorectal cancer (CRC) cells. IR-induced upregulation of intranuclear NF-κB, FUT175 counteracted this effect. Moreover, the combination treatment suppressed cell viability and induced apoptosis. Similar effects were also observed in xenograft tumors. In addition, FUT175 prevented the migration and invasion of cancer cells caused by IR by downregulating the enzymatic activity of MMP-2/-9. In conclusion, FUT175 enhances the anti-tumor effect of radiotherapy through downregulation of NF-κB and reduces IR-induced tumor invasiveness by directly inhibiting MMP-2/-9 in CRC cells. Therefore, the use of FUT175 during radiotherapy might improve the efficacy of radiotherapy in patients with CRC.

## 1. Introduction

Colorectal cancer (CRC) is the fourth leading cause of cancer-related deaths worldwide, with a global incidence of almost 10% in 2012 [1]. Surgery is the standard therapy for patients with CRC; unfortunately, 20% of the patients relapse. Moreover, patients with rectal cancer show a higher rate of postoperative local recurrence, lower disease-free and overall survival rates than patients with colon cancer [2]. To reduce the risk of postoperative recurrence of CRC, perioperative anti-cancer treatments such as chemotherapy and radiotherapy are usually performed. Preoperative chemoradiotherapy, one of the standard treatments for locally advanced rectal cancer [3], reduces the size and stage of tumor, decreases the locoregional recurrence, and improves the sphincter-saving rate in patients [4]. However, some clinical trials have indicated that preoperative chemoradiotherapy does not improve overall survival; the rate of distant recurrence in these conditions remains as high as 15–20%, suggesting that preoperative chemoradiotherapy is not a satisfactory treatment for locally advanced rectal cancer [5]. In this regard, a past report demonstrated that radiotherapy enhances the formation of distant metastases [6]. Therefore, the improvement of the radiosensitivity and/or inhibition of distant metastases may provide a new strategy for locally advanced rectal cancer therapy.

Nuclear factor-kappa B (NF-κB) is a transcriptional factor identified as a protein that binds the promoter of the immunoglobulin kappa chain in B cells. NF-κB is a homo- or heterodimer composed of the p50, p52, p65, c-Rel and RelB subunits. The canonical NF-κB is the heterodimer of the p50 and p65 subunits [7]. NF-κB is involved in many physiological processes such as immune and inflammatory response, apoptosis and cell proliferation [8]. NF-κB is also known to play an important role in regulating oncogenesis, cancer progression and resistance to treatment including chemotherapy and radiotherapy in several types of cancers [9,10,11,12]. In particular, since inhibition of NF-κB activation is a key issue in tumor resistance against ionizing radiation (IR) in CRC cells [13], NF-κB is a promising target for the improvement of radiosensivity in patients with CRC.

Distant metastases are an important issue in radiotherapy for diverse cancers, including CRC. Metastasization is a complex multistep process. Cancer cell invasion requires adhesion of cells to the extracellular matrix and the extracellular matrix degradation by cellular proteases such as matrix metalloproteinases (MMPs). Subsequently, cancer cells migrate into lymph ducts and small vessels to reach metastatic sites [14]. IR increases the migration and invasion capabilities of human cancer cell lines derived from CRC, lung cancer and glioblastoma, together with the upregulation of MMPs such as MMP-2 and MMP-9 [15,16,17]. Several MMP inhibitors, such as marimastat and prinomastat, have been developed, and some trials have shown their positive effect [18]. However, their usefulness is limited, mostly because the use of MMP inhibitors is associated with adverse side effects including musculoskeletal toxicity [19,20].

Nafamostat mesilate (FUT175), a synthetic serine-protease inhibitor, has been widely used for the treatment of pancreatitis, disseminated intravascular coagulation, and systemic inflammatory response syndrome in Japan and Korea [21,22,23]. We have previously reported that FUT175 inhibits NF-κB activation by suppressing IκB phosphorylation and induces caspase-8 mediated apoptosis of pancreatic cancer cells [24]. Moreover, FUT175 inhibits anticancer drugs or IR-induced activation of NF-κB and enhances the chemoradiosensitivity of pancreatic, gastric and gallbladder cancer cells [25,26,27,28]. We also showed that FUT175 decreases MMP-9 activity in pancreatic cancer through NF-κB modulation. However, the effect of FUT175 on irradiated CRC cells is still unknown.

In this study, we investigated whether FUT175 affects IR-induced NF-κB activation and enhances the anti-tumor effect of radiotherapy in CRCs. We also evaluated the effect of FUT175 on the activation of MMP-2 and MMP-9 in irradiated CRC cells.

## 2. Results

### 2.1. Nafamostat Mesilate (FUT175) Prevents NF-κB Activation and Induces Apoptosis in Irradiated Colorectal Cancer (CRC) Cells

**Figure 1:**
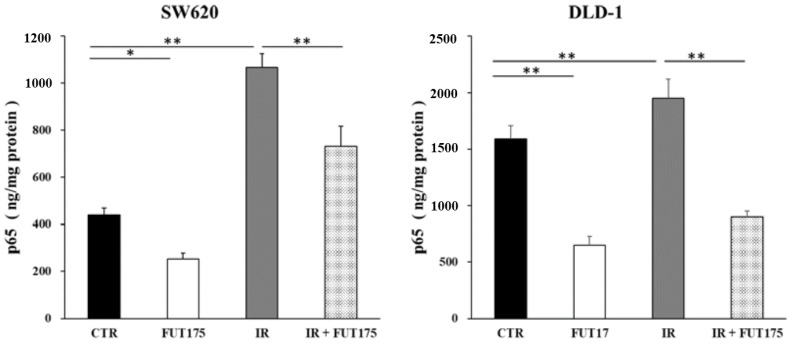
Inhibition of ionizing radiation (IR)-induced activation of nuclear factor kappa B (NF-κB) by nafamostat mesilate (FUT175). Assessment of NF-κB activation (p65 levels) in SW620 and DLD-1 cells by enzyme-linked immunosorbent assay (ELISA). The cells were treated with FUT175 (80 μg/mL) for 3 h prior to irradiation (5 Gy). At 3 h of incubation after the treatment, the levels of NF-κB p65 in the nuclear extracts were measured. p65 concentration was higher in the IR groups than in the control groups (SW620: 1169.4% ± 158.2 vs. 903.3% ± 93.1, DLD-1: 1948.5% ± 169.8 vs. 1589.2% ± 115.7; *p* < 0.01) and decreased in the combination groups (SW620: 447.5% ± 72.2, DLD-1: 899.6% ± 53.7; *p* < 0.01). *, *p* < 0.05; **, *p* < 0.01. All experiments were performed in triplicate.

To investigate whether IR induces the activation of NF-κB in the CRC cells lines SW620 and DLD-1, the intranuclear levels of p65 were assessed by enzyme-linked immunosorbent assay (ELISA). Increased levels of p65 in SW620 and DLD-1 cells were observed after IR (2.4-fold and 1.2-fold increase, respectively). Treatment of the cells with FUT175 prior to IR exposure significantly decreased the levels of p65 in the nucleus, compared with cells irradiated and not treated with FUT175 (Figure 1). This result indicates that FUT175 prevents IR-induced activation of NF-κB in CRC cells.

**Figure 2:**
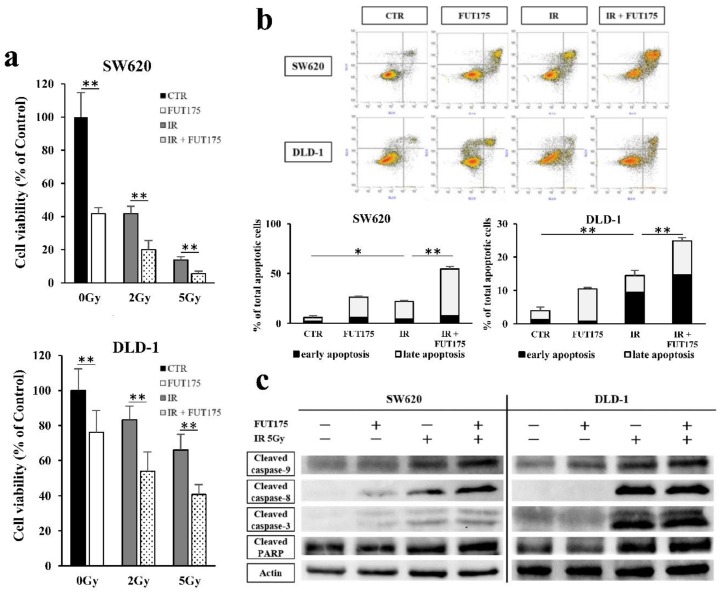
Nafamostat mesilate (FUT175) enhances radiosensitivity and ionizing radiation (IR)-induced cell apoptosis in colorectal cancer (CRC) cells. (**a**) The cells were treated with FUT175 (80 μg/mL) for 3 h prior to irradiation (2 Gy, 5 Gy). At 96 h of incubation after the treatment, the cell viability was measured. The viability of SW620 and DLD-1 cells in the FUT175 groups was significantly lower than that of cells in the control groups (SW620: 41.6% ± 3.8, *p* < 0.01; DLD-1: 76.1% ± 12.5, *p* < 0.01). In the IR groups, cell viability was reduced in a dose-dependent manner. Cell viability in the combination groups was significantly lower than that in the IR groups at each IR dose (SW620, 2 Gy: 20.0% ± 5.5 vs. 41.7% ± 4.5 and 5 Gy: 5.6% ± 1.5 vs. 13.8% ± 1.9, *p* < 0.01; DLD-1, 2 Gy: 54.0% ± 10.8 vs. 83.2% ± 7.8 and 5 Gy: 40.8% ± 5.6 vs. 66.1% ± 8.9, *p* < 0.01). (**b**) The cells were treated with FUT175 (80 μg/mL) for 3 h prior to irradiation (5 Gy). At 72 h of incubation after the treatment, the apoptotic cells were measured by flow cytometry analysis after Annexin/FITC staining. The percentage of early and late apoptotic cells in the combination groups was significantly greater than that in the IR groups (early apoptosis: SW620, 7.5% ± 0.4 vs. 4.5% ± 0.0 and DLD-1, 14.7% ± 0.7 vs. 9.5% ± 1.2, *p* < 0.01; late apoptosis: SW620, 47.2% ± 2.2 vs. 17.5% ± 0.9 and DLD-1, 10.1% ± 0.4 vs. 4.9% ± 0.5, *p* < 0.01). (**c**) The cells were treated with FUT175 (80 μg/mL) for 3 h prior to irradiation (5 Gy). At 24 h of incubation after the treatment, the apoptosis-related proteins were measured by western blot analysis. The levels of cleaved caspase-9/-8/-3, and cleaved PARP in the combination groups were greater than those in the other groups. *, *p* < 0.05; **, *p* < 0.01. All experiments were performed in triplicate.

We next examined the viability of cells after IR and with or without FUT175 treatment by MTT assay. The viability of both CRC cell lines was reduced by radiotherapy in a dose-dependent manner and FUT175 enhanced the anti-proliferative effect of radiotherapy at each IR dose (Figure 2a). Additionally, apoptosis in response to radiation and treatment with FUT175 was analyzed by Annexin V/propidium iodide (PI) staining. IR and FUT175, separately, increased the percentage of apoptotic SW620 and DLD-1 cells (early and late apoptotic cells) to a similar extent (IR: 22% and 14%, FUT175: 26% and 10%, respectively). Interestingly, we observed an additive effect of FUT175 and IR on apoptosis (55% and 25%, respectively, Figure 2b). We confirmed these data in Western blot analyses of apoptosis-related proteins, including the cleaved forms of caspase-9, caspase-8, caspase-3, and poly (ADP-ribose) polymerase (PARP) (Figure 2c). These results suggest that FUT175 enhances the anti-proliferative effects of IR by inducing apoptosis through the inhibition of NF-κB activation in CRC cells.

### 2.2. FUT175 Enhances the Anti-Tumor Effect of Radiotherapy In Vivo

**Figure 3:**
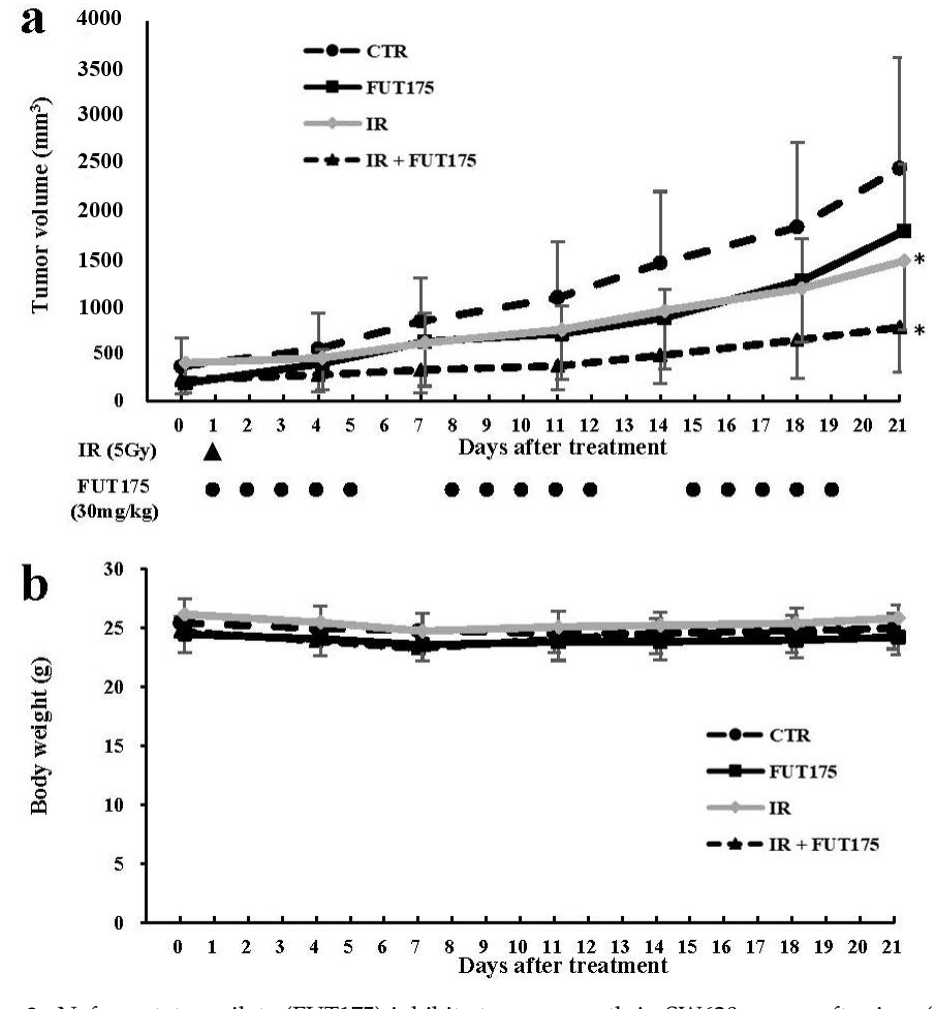
Nafamostat mesilate (FUT175) inhibits tumor growth in SW620 xenograft mice. (**a**) We established a colorectal cancer model by subcutaneously injecting 5 × 10^6^ SW620 cells into the right flank of the animals. Three weeks after injection, the animals were randomized into the following groups: FUT175, intraperitoneally injected with FUT175 (30 mg/kg) five times a week; ionizing radiation (IR), intraperitoneally injected with an equal volume of distilled water five times a week and irradiated once (5 Gy); IR + FUT175, intraperitoneally injected with FUT175 (30 mg/kg) five times a week and irradiated once (5 Gy); CTR, intraperitoneally injected with an equal volume of distilled water five times a week. Mean tumor volume was measured at the indicated times after treatment. The largest tumor volume (mm^3^) measured in the control, FUT175, IR, and combination groups (mean ± standard deviation [SD]) was 2445 ± 1166, 1812 ± 707, 1499 ± 724, and 773 ± 471, respectively. The tumor volume in the combination group was significantly smaller than that in the IR group (*p* < 0.05). (**b**) There were no significant differences in the body weights of the animals among the experimental groups. *, *p* < 0.05.

**Figure 4:**
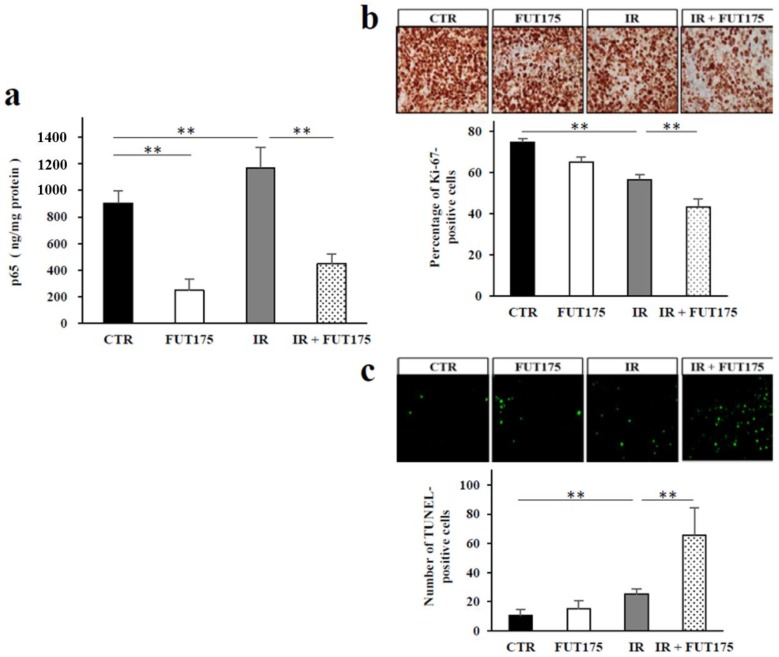
Nuclear factor kappa B (NF-κB) activity and immunohistochemical analysis of cell proliferation and apoptosis in vivo. (**a**) ELISA revealed that the concentration of NF-κB p65 in the nuclear extract of excised tumor tissues in the ionizing radiation (IR) group was significantly higher than that in the control group, and the IR-induced NF-κB activation was inhibited in the combination group (*p* < 0.01). (**b**) The percent of Ki-67-positive cells in the combination group was lower than that in the IR group (43.3% ± 3.9 vs. 56.5% ± 2.6, *p* < 0.01). (**c**) The number of TdT-mediated dUTP nick-end labeling (TUNEL)-positive cells in the combination group was higher than that in the IR group (65.7% ± 18.3% vs. 25.2 ± 3.6, *p* < 0.01). **, *p* < 0.01.

To assess whether FUT175 increases the anti-tumor effect of IR in vivo, a xenograft model was established by injection of SW620 cells into nude mice. Three weeks after injection, the tumor volume in the combination group (IR + FUT175) was lower than that in the IR or FUT175 groups (Figure 3a). No significant differences were observed in the body weight of the animals among the four groups (Figure 3b). We also investigated NF-κB activation in the xenograft tumors from each group. FUT175 inhibited NF-κB activation in mice (Figure 4a) as we had already observed in cultured cells. Histological analysis of the tumors showed that the combination therapy not only decreased the ratio of Ki-67-positive cells, but also increased the number of TdT-mediated dUTP nick-end labeling (TUNEL)-positive cells, as compared to IR monotherapy (Figure 4b,c). These data suggest that FUT175 enhances the radiosensitivity of the cells by inhibiting NF-κB activation and augments IR-induced apoptosis in CRC cells.

### 2.3. FUT175 Prevents the Ionizing Radiation (IR)-Induced Invasiveness of CRC Cells through Inhibition of MMP-2 and -9

**Figure 5:**
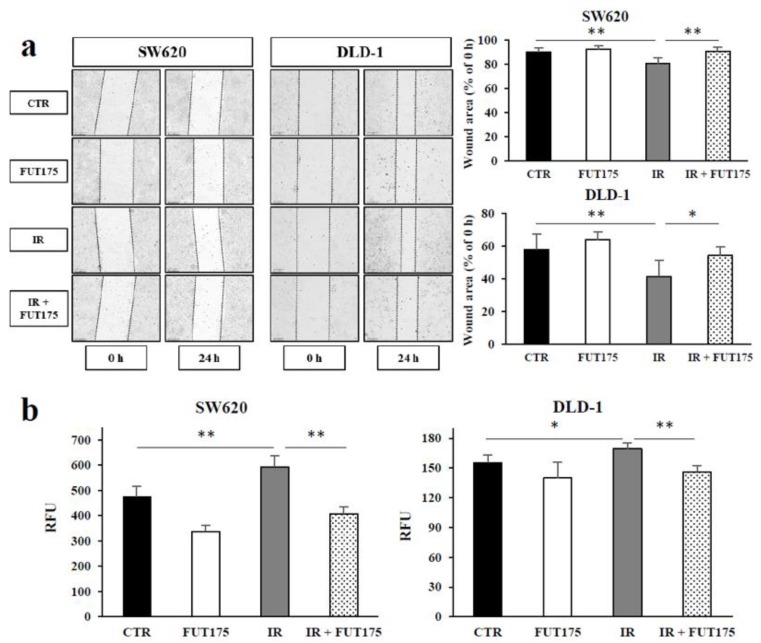
Nafamostat mesilate (FUT175) suppresses ionizing radiation (IR)-induced cell migration and invasion in vitro. (**a**) The cells were treated with FUT175 (80 μg/mL) for 3 h prior to irradiation (5 Gy). Wound healing was measured at 0 and 24 h after the treatment. The wound area of the combination groups was significantly wider than that of the IR groups (SW620: 90.6% ± 3.8 vs. 80.4% ± 5.0, *p* < 0.01; DLD-1: 54.2% ± 5.6 vs. 41.4% ± 9.9, *p* < 0.05). (**b**) Immediately after irradiation (5 Gy), the cells were harvested and reseeded into a 96-well plate at a density of 2 × 10^5^ cells/well and incubated with FUT175 (80 μg/mL) for 24 h. Invading cells were quantified by reading the fluorescence at 480 nm/520 nm. The invasion index of the combination groups was significantly lower than that of the IR groups (SW620: 407.5 ± 25.8 vs. 594.0 ± 43.9, *p* < 0.01; DLD-1: 146.3 ± 6.4 vs. 169.5 ± 5.8, *p* < 0.01). *, *p* < 0.05; **, *p* < 0.01. RFU: Relative fluorescence units. All experiments were performed in triplicate.

To investigate whether FUT175 inhibits IR-induced tumor migration and invasion of CRC cells, we conducted wound healing assays in SW620 and DLD-1 cells. As shown in Figure 5a, the wound area of the irradiated cells was narrowed compared with that in control cells, especially in DLD-1 cells. FUT175 reversed the effect of radiation, suggesting that it prevents the migration of irradiated cells. We also evaluated the effect of FUT175 on IR-induced invasiveness of CRC cells using Matrigel invasion assays. Similarly to the wound healing assays, IR induced CRC cell invasion, while FUT175 suppressed the invasion of irradiated CRC cells (Figure 5b). These data indicate that FUT175 decreases both IR-induced migration and invasion of CRC cells.

**Figure 6:**
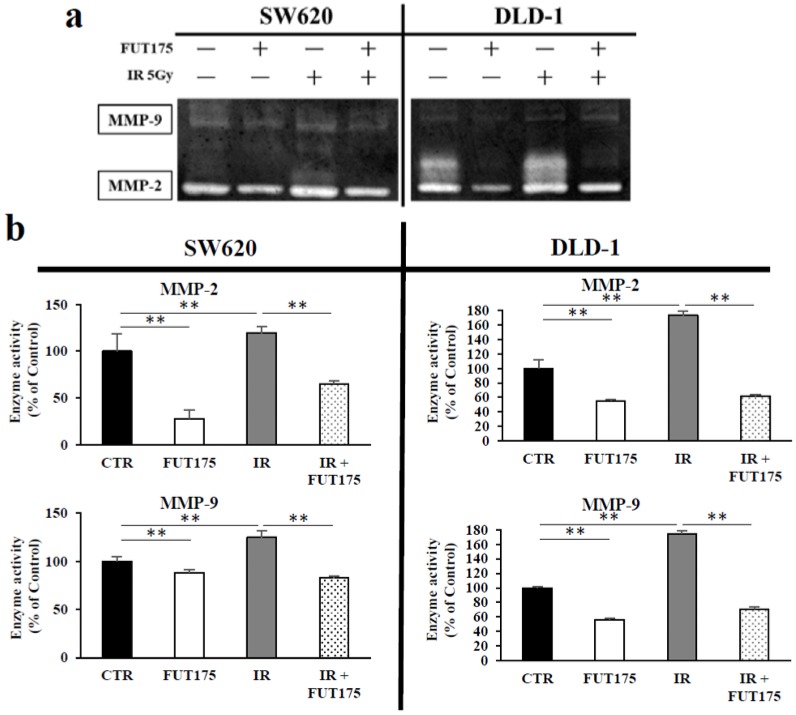
Nafamostat mesilate (FUT175) inhibits the enzymatic activity of matrix metalloproteinase (MMP)-2 and MMP-9 in colorectal cancer (CRC) cells. (**a**) The cells were treated with FUT175 (80 μg/mL) for 3 h prior to irradiation (5 Gy). At 24 h of incubation after the treatment, the conditioned media were measured. Gelatin zymography of CRC cells after 5 Gy ionizing radiation (IR) (or 0 Gy, control) showed that treatment with FUT175 for 24 h decreased the activity of MMP-2 and MMP-9. (**b**) The cells were treated with FUT175 (80 μg/mL) for 3 h prior to irradiation (5 Gy). At 24 h of incubation after the treatment, the conditioned media were measured. Quantitative MMP activity assays showed that the enzymatic activity of MMP-2 and MMP-9 was significantly lower in the combination groups than in the IR groups (MMP-2: SW620, 65.1% ± 3.0 vs. 119.5% ± 6.6 and DLD-1, 61.6% ± 1.6 vs. 173.2% ± 6.0, *p* < 0.01; MMP-9: SW620, 83.1% ± 1.9 vs. 124.8% ± 6.9 and DLD-1, 70.7% ± 2.8 vs. 174.6% ± 4.4, *p* < 0.01). **, *p* < 0.01. All experiments were performed in triplicate.

Next, to investigate the effect of IR and FUT175 on MMP-2 and MMP-9, the expressions of these enzymes in the supernatant of CRC cells were analyzed by gelatin zymography. Although IR increased the enzymatic activity of MMP-2 and MMP-9 secreted from CRC cells, the combination treatment suppressed the activation of these enzymes (Figure 6a). Next, we measured the enzymatic activity of MMP-2 and MMP-9 in the supernatant from CRC cells exposed to IR and treated with or without FUT175. Similarly to the results obtained with gelatin zymography, IR treatment induced a significant increase of the enzymatic activity of MMP-2 and MMP-9 secreted from CRC cells, while FUT175 suppressed this effect (Figure 6b).

**Figure 7:**
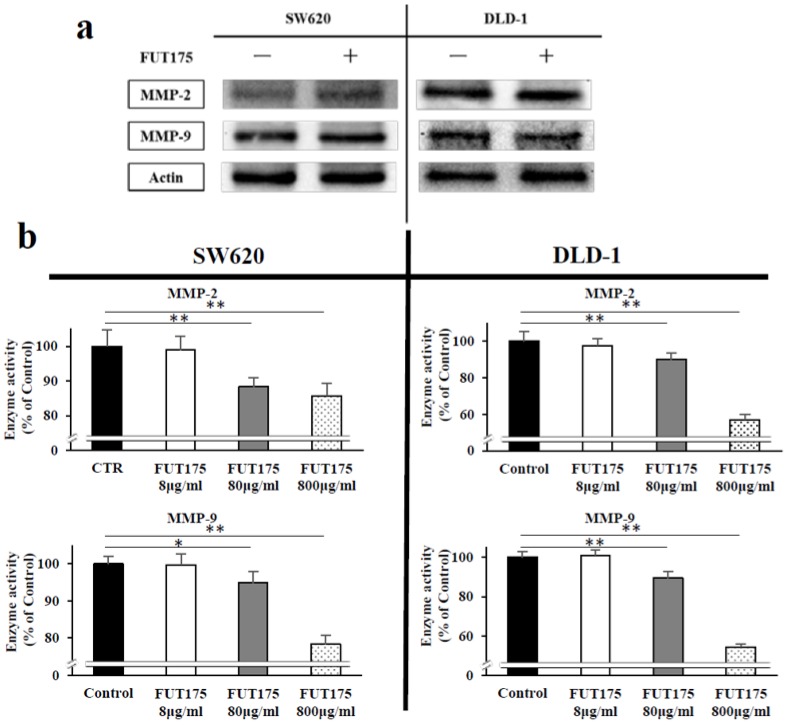
Nafamostat mesilate (FUT175) directly inhibits the activity of matrix metalloproteinase (MMP)-2 and MMP-9. (**a**) The cells were treated with FUT175 (80 μg/mL) for 3 h prior to irradiation (5 Gy). At 24 h of incubation after the treatment, the proteins of MMP-2 and MMP-9 were measured by western blot analysis. FUT175 did not significantly alter the protein expression of MMP-2 and MMP-9. (**b**) The enzymatic activity of MMP-2 and MMP-9 was measured immediately after FUT175 treatment (control, or 8, 80, 800 μg/mL) by quantitative MMP activity assay. The activity of MMP-2 and MMP-9 was significantly and dose-dependently inhibited by FUT175. *, *p* < 0.05; **, *p* < 0.01. All experiments were performed in triplicate.

The expression of MMP-2 and MMP-9 was also analyzed by Western blot analyses: no significant differences were observed between the control and FUT175 group (Figure 7a). On the other hand, when FUT175 was added to cell extracts from CRC cells, the enzymatic activity of MMP-2 and MMP-9 decreased in a dose-dependent manner (Figure 7b). These results suggest that FUT175 suppresses the IR-induced invasion ability of CRC cells through direct inhibition of the enzymatic activity of MMP-2 and MMP-9.

## 3. Discussion

Radiotherapy is widely used as preoperative neoadjuvant or postoperative adjuvant therapy for rectal cancer and as a palliative treatment for recurrent or metastatic cancer. However, the current outcome from radiotherapy is not always satisfactory and the augmentation of tumor radiosensitivity is desirable. NF-κB is a promising target in this regard. Several studies have reported that NF-κB inhibition enhances radiosensitivity: for example, NF-κB inhibition, through modulation of IκB, enhances IR-induced apoptosis in head-and-neck cancer and colon cancer cells [29,30]. Several agents, such as bortezomib [31], sorafenib [32], curcumin [33] and genistein [34], also lead to better outcomes in various cancers by enhancing the radiosensitivity through NF-κB inhibition. However, the use of NF-κB inhibitors is associated with serious adverse side-effects. In this study, we found that FUT175 increases cell apoptosis in irradiated CRC cells via inhibition of NF-κB activation through the caspase signaling (Figure 1 and Figure 2). Our results also demonstrated that FUT175 enhances the anti-tumor effect of IR in a CRC xenograft model without obvious body weight loss (Figure 3). These data suggest the possible benefit of the clinical use of this agent. In Japan, FUT175 has been approved as therapy for pancreatitis and disseminated intravascular coagulation since 1986, and serious adverse events associated with its use have not been reported [22,23]. On the other hand, in clinical, FUT175 is administrated by intravenous injection. As the half-life of FUT175 is short in the human body, it is difficult to keep an effective blood concentration for tumors, suggesting that clinical application of FUT175 for treatment of CRC patients requires a ingenuity of administration route. Indeed, our previous phase I and II clinical study, which analyzed the combination effect of FUT175 and gemcitabine on unresectable pancreatic cancer patients, employed the insertion of arterial catheter into the common hepatic artery through a side hole in the celiac artery for injection of FUT175, and continuously administered the agent. The clinical study showed not only the improvement of median survival time in patients treated with a combination of FUT175 and gemcitabine, but also no additive adverse events in them compared to patients treated with gemcitabine alone [35,36]. Therefore, FUT175 seems to be a safely applicable, and clinically promising approach for improving radiosensitivity of CRC patients.

We demonstrated the preventive effect of FUT175 on the irradiation-induced NF-κB activation in CRC cells. FUT175 is also known to inhibit the chemotherapy-induced translocation of NF-κB to nuclear in several cancer cell lines including pancreatic, gastric and gallbladder cancer cells [25,26,27], indicating that pre-treatment of FUT175 can suppress the induction of NF-κB in various cell lines which were treated with anti-cancer therapy. On the other hand, effect of post-treatment of FUT175 on NF-κB in cancer cells is still unknown.

We used two cell lines derived from colon cancer but not from rectal cancer for evaluating the radiosensitivity of colorectal cancer in this study. From a clinical point of view, the colon and rectal cancers are treated as distinct entities. Some evidence also supports the difference of molecular pathways in carcinogenesis between colon and rectal cancers [37,38]. However, Spitzner et al. showed no significant difference in chemoradiosensitivity between colon and rectal cancer cell lines [39]. Indeed, previous studies that evaluated the effects of radiotherapy for colorectal cancer have frequently used colon cancer cell lines [31,40,41]. Therefore, our results appear to retain the translational aspect about improving the radiosensitivity to colorectal cancers.

Previous findings have indicated that IR increases tumor cell migration and invasion through complex effects on tumor microenvironment, intercellular junctions, extracellular matrix junctions, protease secretion, and epithelial mesenchymal transition [42]. Metastases could be caused by the enhanced migratory properties of the cancer cells surviving IR. Our results indicate that IR promotes cell migration and invasion in CRC cells. We also found that FUT175 suppresses IR-induced cell migration and invasion, suggesting that FUT175 is an effective drug for inhibition of metastasis formation in IR-treated CRC cells (Figure 5a,b).

MMPs are a family of zinc-dependent endopeptidases involved in the degradation of various proteins in the extracellular matrix [43]. MMPs promote cell proliferation, migration, and differentiation, and could play a role in cell apoptosis, angiogenesis, tissue repair, and immune response [44]. MMP expression is deregulated in cancer, and elevated MMP levels are associated with tumor progression and invasiveness. Among MMPs, MMP-2 and MMP-9 can degrade type IV collagen, which is a major component of the basement membrane. Thus, these MMPs play a central role in cancer cell invasion and metastasis [45]. It has been reported that poor prognosis in several patients with cancer correlates with the increased activity of MMP-2 and MMP-9 [46,47]. Moreover, preoperative radiotherapy leads to a significant increase in the levels of MMP-2 and MMP-9 in rectal cancer tissues without their correspondent increase in normal mucosa [48]. Similarly, we found that the increased invasiveness after IR is associated with the increased activity of MMP-2 and MMP-9 in CRC cells. Notably, our results indicate that FUT175 directly inhibits the enzymatic activity of MMP-2 and MMP-9. The inhibition of these MMPs might be one of the mechanisms through which FUT175 exerts its inhibitory effect on IR-induced invasion.

We previously demonstrated that FUT175 suppresses the activity of MMP-9 in pancreatic cancer cells by regulating NF-κB [49]. Interestingly, the current study shows that FUT175 treatment not only inhibits the enzymatic activity of MMP-9, but also that of MMP-2 in irradiated CRC cells, while it does not significantly change their protein levels (Figure 6 and Figure 7). In addition, FUT175 decreases the activity of MMP-2 in a dose-dependent manner, confirming the inhibition of MMP-2 activation by this agent. This result is supported by the data showing that *MMP-2* is not directly regulated by NF-κB [50]. On the other hand, *MMP-9* is one of the genes regulated by NF-κB [51]. Our results indicate that FUT175 does not reduce the expression of MMP-9 at the protein level in spite of the reduction of intranuclear p65 levels (Figure 1 and Figure 7a), suggesting that NF-κB may not be the major transcriptional factor for MMP-9 regulation in the CRC cell lines we investigated. In this regard, a number of binding sites for transcriptional factors have been identified in the promoter region of *MMP-9*, whose expression is regulated by various transcriptional factors including NF-κB, AP-1 and Sp-1 [52]. Inhibition of Sp-1 transcriptional activity downregulates MMP-9 expression in CRC cells [53]. Some MMPs, including MMP-2 and MMP-9, are produced as inactive pro-enzymes from cancer cells and are then activated by proteolytic cleavage. Pro-MMP-2 and pro-MMP-9 are known to be activated by enzymatic cascades consisting of multiple enzymes, including serine proteases such as plasmin and urokinase plasminogen activator [43]. Because FUT175 is an artificial serine protease inhibitor, the inhibitory effect of this drug on the enzymatic activity of MMP-2 and MMP-9 may be due to defective activation of the pro-enzymes by serine proteases.

## 4. Materials and Methods

### 4.1. Cell Culture

The human CRC cell lines SW620 and DLD-1 were obtained from American Type Culture Collection (Manassas, VA, USA). SW620 cells were maintained in Dulbecco’s modified Eagle’s Medium (Wako Pure Chemical Industries, Ltd., Osaka, Japan) containing 10% fetal bovine serum (Gibco BRL, Grand Island, NY, USA) and 1% penicillin/streptomycin (Gibco BRL). DLD-1 cells were maintained in Roswell Park Memorial Institute 1640 medium (Wako Pure Chemical Industries, Ltd.) containing 10% fetal bovine serum (FBS) and 1% penicillin/streptomycin. Cells were cultured at 37 °C in a humidified atmosphere of 5% CO_2_.

### 4.2. Reagents

FUT175 was gifted by Torii Pharmaceutical Co., Ltd. (Tokyo, Japan), dissolved in sterile distilled water (5 mg/mL) and stored at −20 °C.

### 4.3. Ionizing Radiation

In vitro, the cells were irradiated with the indicated doses using the X-ray irradiator MBR-1520R-3 (HITACHI Engineering & Services Co., Ltd., Tokyo, Japan). In vivo, the tumor was irradiated (5 Gy) using the above-mentioned X-ray irradiator. The mice were immobilized in a customized harness allowing exposure of the right flank, whereas the remainder of the body was shielded by an X-ray protector (HAGOROMO; Maeda Co., Tokyo, Japan).

### 4.4. Antibodies

Monoclonal antibodies specific to cleaved caspase-9, cleaved caspase-8, cleaved caspase-3, and cleaved PARP, as well as polyclonal antibodies specific to MMP-2 and MMP-9 were obtained from Cell Signaling Technology (Beverly, MA, USA). The monoclonal anti-β-actin antibody was purchased from Sigma (St. Louis, MO, USA) and the monoclonal anti-Ki-67 antibody was purchased from Dako (Produktionsvej, Glostrup, Denmark).

### 4.5. In Vitro Experiments’ Treatment Groups

CRC cells were treated with FUT175 (80 μg/mL; nafamostat mesilate group, FUT175), ionizing radiation (ionizing radiation group, IR), both FUT175 (80 μg/mL) and ionizing radiation (combination group, IR + FUT175), or vehicle-only (control group, CTR) for the appropriate time. Cells of the IR and combination groups received 2 or 5 Gy IR for the cell proliferation assay, and 5 Gy IR for the other analyses. In the combination group, the cells were treated with nafamostat mesilate for three hours before IR.

### 4.6. Cell Proliferation Assays

The cells were seeded into 96-well plates (1 × 10^3^ cells in each well) and treated as indicated for 96 h. Cell viability was assessed using the CellTilter 96^®^ Aqueous One Solution Cell Proliferation Assay (Promega; Madison, WI, USA), according to the manufacturer’s instructions.

### 4.7. Apoptosis Analysis by Flow Cytometry

The cells (1 × 10^6^ cells) were seeded and treated with each regimen for 72 h. Apoptosis was assessed using an Annexin V/fluorescein isothiocyanate (FITC) kit (Miltenyi Biotec, Sunnyvale, CA, USA), according to the manufacturer’s instructions. The stained cells were analyzed with an Attune NxT Flow Cytometer (Thermo Fisher Scientific; Waltham, MA, USA).

### 4.8. Western Blot Analysis

The cells (5 × 10^6^ cells) were seeded into a 100-mm dish and treated as indicated. Whole cell lysates were prepared as previously described [54], separated by sodium dodecyl sulfate polyacrylamide gel electrophoresis and transferred to nitrocellulose membranes. After incubation in the primary antibodies (1:1000 dilution) overnight, the membranes were incubated with the appropriate secondary antibodies (1:10,000 dilution, Histofine; Nichirei, Tokyo, Japan). Immunocomplexes were detected using the immunostar LD chemiluminescent reagent (WAKO chemical, Tokyo, Japan) and a Chemi Doc XRS+ system and Image Lab software (Bio-Rad, Hercules, CA, USA).

### 4.9. Quantitative Analysis of NF-κB Activity

The levels of NF-κB p65 in the nuclear extracts were measured. Nuclear extracts from cells or tissues were prepared using a nuclear extraction kit (Active Motif; Carlsbad, CA, USA) according to the manufacture’s protocol and assayed using a Trans AM p65 NF-κB Kit (Active Motif), which is an ELISA-based method designed to quantify the activation of the NF-κB p65 subunit, according to the manufacture’s protocol.

### 4.10. Wound-Healing Assays

The cells (1 × 10^6^ cells) were seeded in 6-well plates to form a confluent monolayer. Cells in the center of the well were scratched using a sterile 200 μL pipette tip; the cells in each well were then treated as described; photos were taken at 0 and 24 h using an IX71-Olympus inverted microscope (Olympus; Tokyo, Japan) at 10× magnification. The wound-healing index is expressed as a percentage of the wound area at 24 h divided by the wound area at 0 h.

### 4.11. Invasion Assays

The invasive ability of CRC cells was assessed using a CytoSelect^TM^ 96-well Cell Invasion Assay kit (Cell Biolabs; San Diego, CA, USA) according to the manufacturer’s instructions.

### 4.12. Gelatin Zymography

The expression of MMP-2 and MMP-9 in the culture media was analyzed using a gelatin-zymography kit (Primary Cell; Hokkaido, Japan) according to the manufacturer’s instructions. After the indicated treatment for 24 h, the conditioned medium was concentrated 10-fold using Amicon Ultra-4 Filter Units (Merck; Darmstadt, Germany). Each sample was subjected to electrophoresis on a gelatin-containing gel. The gels were washed and incubated for 40 h in an incubation buffer at 37 °C. After Coomassie blue staining, the gels were scanned.

### 4.13. Matrix Metalloproteinase (MMP) Activity Assays

MMP activity was analyzed using the Amplite Universal Fluorimetric MMP Activity Assay Kit (AAT Bioquest; Sunnyvale, CA, USA) according to the manufacturer’s instructions.

### 4.14. Animal Models

Five-week-old male nude mice (BALBc nu/nu), purchased from CLEA Japan Inc. (Tokyo, Japan), were housed under specific pathogen-free conditions at the Laboratory Animal Facility of the Jikei University School of Medicine. All protocols complied with institutional guidelines and were approved by the Institutional Animal Care and Use Committee of the Jikei University School of Medicine (2015-014). We established a colorectal cancer model by subcutaneously injecting 5 × 10^6^ SW620 cells into the right flank of the animals. Three weeks after injection, the animals were randomized into the following groups: FUT175 (*n* = 7), intraperitoneally injected with FUT175 (30 mg/kg) five times a week; IR (*n* = 7), intraperitoneally injected with an equal volume of distilled water five times a week and irradiated once (5 Gy); IR + FUT175 (*n* = 7), and CTR (*n* = 7). Body weight was measured three times a week as an indicator of systemic toxicity. Tumor volume was estimated from two-dimensional tumor measurements with the formula: *V* = length (mm) × width^2^ (mm^2^)/2. A subset of the animals was sacrificed 7 days after the treatments (*n* = 6 each), and tumors were dissected for ELISA and histologic analyses.

### 4.15. Immunohistochemical Staining and TdT-Mediated dUTP Nick-End Labeling (TUNEL) Assays

Paraffin sections of tumor tissues were stained immunohistochemically using a Ki-67 antibody (1:300 dilution) and visualized using the BenchMark-XT system (VENTANA Medical System; Tucson, AZ, USA). The TUNEL assay was performed using an In Situ Cell Death Detection Kit, Fluorescein (Roche Diagnostics, Indianapolis, IN, USA) to evaluate the induction of apoptosis. The percentage of Ki-67-positive cells was microscopically examined in three random high-power fields at 400× magnification from three tumors. The number of TUNEL-positive cells was counted at 200× magnification.

### 4.16. Statistical Analysis

Data are expressed as the mean ± standard deviation (SD). A non-paired *t*-test (two-tailed) was used to compare the two groups. One-way analysis of variance was used when comparing multiple groups followed by the Turkey’s post-hoc test. All *p*-values were considered statistically significant when <0.05.

## 5. Conclusions

Our study highlights the multiple functions of FUT175 for the improvement of radiosensitivity in CRC. We demonstrated for the first time that FUT175 can inhibit the enzymatic activity of MMP-2 and MMP-9 at a post-translational level. Our findings suggest that the use of FUT175 during radiotherapy might be a new therapeutic approach to improve the efficacy of radiotherapy in patients with CRC.
