# Correction: Hiroshi Sugano et al. Nafamostat Mesilate Enhances the Radiosensitivity and Reduces the Radiation-Induced Invasive Ability of Colorectal Cancer Cells. *Cancers* 2018, *10*, 386

**DOI:** 10.3390/cancers11030335

**Published:** 2019-03-08

**Authors:** Hiroshi Sugano, Yoshihiro Shirai, Takashi Horiuchi, Nobuhiro Saito, Yohta Shimada, Ken Eto, Tadashi Uwagawa, Toya Ohashi, Katsuhiko Yanaga

**Affiliations:** 1 Department of Surgery, The Jikei University School of Medicine, 3-25-8, Nishi-Shinbashi, Minato-ku, Tokyo 105-8461, Japan; shirai@jikei.ac.jp (Y.S.); horiuchi@jikei.ac.jp (T.H.); h24dr-saito@jikei.ac.jp (N.S.); etoken@jikei.ac.jp (K.E.); uwatadashi@msn.com (T.U.); kyanaga@jikei.ac.jp (K.Y.); 2 Division of Gene Therapy, Research Center for Medical Science, The Jikei University School of Medicine, 3-25-8, Nishi-Shinbashi, Minato-ku, Tokyo 105-8461, Japan; shimada_y@jikei.ac.jp (Y.S.); tohashi@jikei.ac.jp (T.O.); 3 Division of Medical Oncology and Hematology, Department of Internal Medicine, The Jikei University School of Medicine, 3-25-8, Nishi-Shinbashi, Minato-ku, Tokyo 105-8461, Japan

The authors would like to make a correction to their published paper [1].

There was a mistake in the original version of the article in the legend of Figure 1. The data are in vivo data of SW620 cells (Figure 4a) and not in vitro data. The authors wish to replace the 5th sentence in legend of Figure 1 with:

“p65 concentration was higher in the IR groups than in the control groups (SW620: 1067.5 ± 55.9 vs. 440.1 ± 30.5, DLD-1: 1948.5 ± 169.8 vs. 1589.2 ± 115.7; *p* < 0.01) and decreased in the combination groups (SW620: 731.8 ± 85.0, DLD-1: 899.6 ± 53.7; *p* < 0.01).”

In addition, there was a mistake on page 4, section 2.2, line 2:

“Three weeks after injection,” should be “Three weeks after treatment,”.

The changes do not affect the scientific results.

The rest of the manuscript does not need to be changed. The authors would like to apologize for any inconvenience caused. The manuscript will be updated, and the original will remain available on the article webpage.
